# On the Rule of Mixtures for Predicting Stress-Softening and Residual Strain Effects in Biological Tissues and Biocompatible Materials

**DOI:** 10.3390/ma7010441

**Published:** 2014-01-16

**Authors:** Alex Elías-Zúñiga, Karen Baylón, Inés Ferrer, Lídia Serenó, Maria Luisa Garcia-Romeu, Isabel Bagudanch, Jordi Grabalosa, Tania Pérez-Recio, Oscar Martínez-Romero, Wendy Ortega-Lara, Luis Ernesto Elizalde

**Affiliations:** 1 Centro de Innovación en Diseño y Tecnología, Tecnológico de Monterrey, Campus Monterrey, E. Garza Sada 2501 Sur, Monterrey 64849, NL, Mexico; E-Mails: kl.baylon.phd.mty@itesm.mx (K.B.); tany.nohemi@gmail.com (T.P.-R.); oscar.martinez@itesm.mx (O.M.-R.); wlortega@gmail.com (W.O.-L.); 2 Department of Mechanical Engineering and Industrial Construction, University of Girona, Maria Aurelia Capmany 61, Girona 17071, Spain; E-Mails: ines.iferrer@udg.edu (I.F.); lidia.sereno@udg.edu (L.S.); mluisa.gromeu@udg.edu (M.L.G.-R.); isabel.bagudanch@udg.edu (I.B.); jordi.grabalosa@udg.edu (J.G.); 3 Centro de Investigación en Química Aplicada, Blvd. Enrique Reyna Hermosillo 140 Saltillo, Coahuila CP25250, Mexico; E-Mail: elizalde@ciqa.mx

**Keywords:** stress-softening effects, biomaterial residual strains, biological tissues, rule of mixtures, pseudo-elasticity theory

## Abstract

In this work, we use the rule of mixtures to develop an equivalent material model in which the total strain energy density is split into the isotropic part related to the matrix component and the anisotropic energy contribution related to the fiber effects. For the isotropic energy part, we select the amended non-Gaussian strain energy density model, while the energy fiber effects are added by considering the equivalent anisotropic volumetric fraction contribution, as well as the isotropized representation form of the eight-chain energy model that accounts for the material anisotropic effects. Furthermore, our proposed material model uses a phenomenological non-monotonous softening function that predicts stress softening effects and has an energy term, derived from the pseudo-elasticity theory, that accounts for residual strain deformations. The model’s theoretical predictions are compared with experimental data collected from human vaginal tissues, mice skin, poly(glycolide-co-caprolactone) (PGC25 3-0) and polypropylene suture materials and tracheal and brain human tissues. In all cases examined here, our equivalent material model closely follows stress-softening and residual strain effects exhibited by experimental data.

## 1. Introduction

It is well-known that most of the constitutive relations available in the literature can not predict most biological material behaviors well, such as the multiaxial behavior of muscles, the softening of biological tissues, damage and healing, among others, because of the molecular and cellular contributions to the behavior at the tissue and organ levels, soft tissue anisotropy, transverse isotropy by tendons and ligaments, cylindrical orthotropy by arteries and complex symmetries by planar tissues [1,2]. Therefore, if one wants to have better prediction of experimental observations, the different material response effects have to be included by the material constitutive model under consideration. In an attempt to have a better prediction of experimental material observations, Holpzafel and coworkers suggested an additive split of the isochoric strain-energy density function into isotropic and anisotropic effects [1]. Sansour carried out an analysis that shows that the volumetric-isochoric split of the material energy density function can be justified on the basis of certain physical observations that are independent of the multiplicative decomposition of the deformation gradient. The analysis shows that care must be exercised in the case of the anisotropic material description in order not to violate certain physical requirements [3]. Sacks assumed that the total strain energy of the chemically treated tissue is the sum of the fiber and matrix components. He used the rule of mixtures with a fiber volume fraction to develop a material model in which the isotropic strain energy function was found to closely follow the matrix stress-strain data [4]. By considering the materials as a hardening material and a perfectly plastic material, Kim *et al*. used the rule of mixtures to predict the mechanical response of composites with homogeneously distributed particles in which the elastic and plastic properties were found as a function of the volumetric fraction of soft and hard particles [5].

Based on these energy models on which the energy is split into two parts, it is clear that an equivalent energy model that considers the matrix (isotropic part) and the fiber (anisotropic part) effects could be used to predict the material behavior that is exhibited by biological tissues and biocompatible materials [6]. Therefore, the aim of this paper focuses on the development of an equivalent material model to predict the response of biocompatible materials, subjected to uniaxial extension or compression loads, in which the total strain energy density is split into the isotropic part, related to the matrix component, and the anisotropic energy contribution, related to the fiber part, by using the rule of mixtures. Furthermore, we will show that the usage of the amended non-Gaussian strain energy density expression of the well-known average-stretch full-network model for rubber elasticity, as the isotropic energy part, provides expressions that describe the qualitative and quantitative response behavior exhibited by the biocompatible materials considered here well.

The paper has been organized as follow. In Section 2, we introduce a brief review of the required equations that describe finite deformations of hyperelastic materials. In Section 3, we introduced an equivalent strain energy density representation form that combines, by using the rule of mixtures, the isotropic and the anisotropic energy material parts. In Section 4, we have derived the corresponding stress-stretch constitutive equations that are based on the amended non-Gaussian strain energy density model, a non-monotonous stress-softening function and a residual strain effects material model that is derived from the pseudo-elasticity theory concepts. Furthermore, we have included the Dorfmann and Ogden material model with slight modifications to capture Mullins and permanent set effects. Comparison of the model’s prediction with experimental data is done in Section 5. Finally, some conclusions related to theoretical predictions and experimental data are addressed in Section 6.

## 2. Basic Equations of Finite Deformations

Since biocompatible materials tend to exhibit large deformations, in this section, we introduce some basic definitions related to finite deformations that are needed to characterize the material behavior. Let Ω be a fixed reference configuration of a body, and use the notation χ : Ω → ℝ^3^ to denote the body deformation, which transforms a material point, **X** ∈ Ω, to the place x = χ(**X**) ∈ Ω*_c_*, the deformed configuration. By definition, the deformation gradient and the local volume ratio are given by **F**(**X**) = ∂χ(**X**)/∂**X** and *J*(**X**) = *det***F** > 0, respectively. By following [7], the deformation gradient can be rewritten as:

$$\bar{F}=\frac{F}{J^{1/3}}$$ (1)

which describes an isochoric deformation, since *det* $\bar{F}=1$. Let us define the isochoric left Cauchy–Green deformation tensor as:

$$\bar{C}={\bar{F}}^{T}\bar{F}=\frac{C}{I_{3}^{1/3}}$$ (2)

in which the relation *J*^2^ = *I*_3_ has been used [3]. Here, the principal invariants, *I_k_* of C, are defined by:

$$\begin{array}{ccc} I_{1}=\text{trC},\; & I_{2}=\frac{1}{2}[I_{1}^{2}-\text{tr}({\text{C}}^{2})], & I_{3}= \end{array}\text{detC}$$ (3)

where tr is the trace operation. Furthermore, the Cauchy–Green deformation tensor B ≡ **FF***^T^* has the form:

$$\text{B}=\lambda_{1}^{2}{\text{e}}_{11}+\lambda_{2}^{2}{\text{e}}_{22}+\lambda_{3}^{2}{\text{e}}_{33}$$ (4)

where e*_jk_* ≡ e*_j_* ⊗ e*_k_* and e*_i_* are the associated orthonormal principal directions and λ*_i_* denote the principal stretches in a common orthonormal frame *φ* = {O; e*_k_*}. Furthermore, the magnitude of the strain intensity at a material point, X, denoted by *m*, is defined by $m\equiv \sqrt{B\cdot B}=\sqrt{{\text{trB}}^{2}}$. Thus, the magnitude of the strain intensity, *m*, can be computed from:

$$m=\sqrt{I_{1}^{2}-2I_{2}}$$ (5)

In the undeformed state B = 1, the identity tensor and $m=\sqrt{3}$; otherwise, $m>\sqrt{3}$ for all isochoric deformations [8].

## 3. Equivalent Strain Energy Density Model

The main motivation on deriving an equivalent strain energy density model not only comes from the ideas previously developed by the aforementioned research works in which the material energy density were split into two parts, but also from the experimental findings obtained in samples of vulcanized natural rubber during uniaxial deformation tests in which the usage of synchrotron X-rays allowed for the determination of the isotropic and anisotropic energy contributions to the material response behavior [9]. Therefore, it is clear that a material model must involve both energy contributions. Here, we shall assume that the total strain energy density, *W_T_*, is given, in accordance with the basic rule of mixtures, as:

$$W_{T}=\left(1-f\right)W_{\text{iso}}\left(I_{1}\right)+fW_{\text{aniso}}\left(I_{4i},I_{5i}\right)$$ (6)

where *W*_iso_(*I*_1_) is the strain energy density related to the isotropic material behavior, *W*_aniso_ (*I*_4_*_i_*, *I*_5_*_i_*) is the anisotropic strain energy density part, *I_4i_* and *I*_5_*_i_* represent the square of the stretch of the *i*-th fiber family, defined as:

$$\begin{array}{cc} I_{4i}=a_{i}\cdot C\cdot a_{i}, & I_{5i}=a_{i}\cdot C^{2}\cdot a_{i} \end{array}$$ (7)

*f* represents the equivalent anisotropic volumetric fraction contribution to the total material energy density and the fiber directions are given by the vectors a*_i_* = *x*_1_*_i_*e_1_ + *x*_2_*_i_*e_2_ + *x*_3_*_i_*e_3_ in the initial configuration, *x_ji_* are the direction cosines of the *i*-th fiber. Of course, several forms can be assumed for W_iso_(*I*_1_) and *W*_aniso_(*I*_4_*_i_*, *I*_5_*_i_*); see, for instance, [1,10–12]. In this work, we shall use the amended non-Gaussian strain energy density to characterize the isotropic contribution (matrix) to the total material energy [11], which is given as:

$$W_{\text{iso}}(I_{1})=\mu \left[N\left(\beta \lambda_{r}+\text{ln}\left(\frac{\beta }{\text{sinh}\beta }\right)\right)\right]-\text{ln}\left(\frac{\beta }{\lambda_{r}}\right)+c$$ (8)

where λ*_r_* is the relative chain stretch:

$$\lambda_{r}=\frac{\lambda_{\text{chain}}}{\lambda_{L}}$$ (9)

$\lambda_{L}=\sqrt{N}$ represents the fully extended chain stretch, *N* is the chain number of rigid links, each of length *l*, λ_chain_ is the chain deformation, which can be computed from:

$$\lambda_{\text{chain}}\equiv \sqrt{\frac{I_{1}}{3}}$$ (10)

*ß* ≡ ℒ^−1^ (λ*_r_*) is the inverse of the Langevin function ℒ(*β*), which is defined as:

$$\lambda_{r}=ℒ(\beta )\equiv \text{coth}\beta -\frac{1}{\beta }$$ (11)

*μ* is the material shear modulus and *c* is an energy constant [11,12]. For the case of *W*_aniso_(I_4_*_i_*, I_5_*_i_*), we assume that this energy term (fiber part) can be equivalently written as a function of *W_T_* and *W*_iso_(*I*_1_) as:

$$W_{\text{aniso}}(I_{4},I_{5})=\frac{W_{T}-(1-f)W_{\text{iso}}(I_{1})}{f}$$ (12)

We next follow a procedure similar to the one developed in [13] in which:

$$W_{\text{aniso}}(I_{4i},I_{5i})\equiv \infty_{i}f_{i}\left(A_{1}(\lambda_{fiberi}^{2}-1)+A_{2}{\left(\lambda_{fiberi}^{2}-1\right)}^{2}-2A_{1}\text{ln}\sqrt{J_{i}}\right)$$ (13)

$$\lambda_{fiberi}^{2}=\lambda_{1i}^{2}x_{1i}^{2}+\lambda_{2i}^{2}x_{2i}^{2}+\lambda_{3i}^{2}x_{3i}^{2}$$ (14)

has been *isotropized* to the form:

$$W_{\text{aniso}}\equiv f\left(\frac{A_{1}}{3}(I_{1i}-3)+\frac{A_{2}}{9}{\left(I_{1i}-3\right)}^{2}-\frac{2A_{1}}{3}\text{ln}\sqrt{I_{3i}}\right)$$ (15)

since the fiber direction cosines have been assumed to have the following possible orientations, (1*,* 1*,* 1), (*−*1*,* 1*,* 1), (1*, −*1*,* 1) and (*−*1*, −*1*,* 1), and *A*_1_ and *A*_2_ are energy density fitting parameters. Other forms for the strain energy density are possible, but we prefer to use expression Equation (15), since its derivation is based on the average orientation with respect to the principal stretch directions of the eight-chain model [13]. Of course, other fiber orientations can be considered to describe the composite material’s behavior, as pointed out by Cantournet and co-workers in [13]. Thus, the substitution of Equation (15) into Equation (6) gives the total equivalent strain energy density expression that can be used to model hyperelastic materials:

$$W_{T}=(1-f)W_{\text{iso}}(I_{1})+f\left(\frac{A_{1}}{3}(I_{1i}-3)+\frac{A_{2}}{9}{\left(I_{1i}-3\right)}^{2}-\frac{2A_{1}}{3}\text{ln}\sqrt{I_{3i}}\right)$$ (16)

## 4. Constitutive Material Models

**Figure 1.**
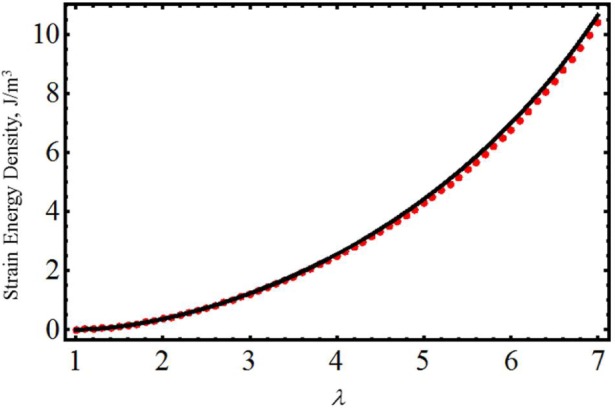
Comparison between the experimental strain energy density and the theoretical predictions obtained from Equations (8) and (16). Here, the red dots represent the experimental data, while the black solid line describes the theoretical predictions. The experimental data was adapted from [9].

Before we use Equation (16) to derive constitutive models to characterize biological materials, let us first assess the accuracy of Equation (16) by using the experimental data collected by Toki and coworkers [9]. The red dots of Figure 1 represent the experimental strain energy density data obtained by integrating the stress *versus* stretch data shown in Figure 1 of [9]. In our Figure 1, the black solid line represents the predicted strain energy density found from Equations (8) and (16), with material parameter values of *A*_1_ = 0 MPa*, A*_2_ = 0.0001 MPa, *c* = −0.1880 J/m^3^, *μ* = 0.475 MPa and *N* = 28.13. As expected, the value of the equivalent anisotropic volumetric fraction contribution is fixed at *f* = 0.25. This value of *f* agrees with the percentage of the anisotropic material found in [9] via *in situ* synchrotron wide-angle X-ray diffraction (WAXD) at each strain during loading and unloading of the material samples. Notice from Figure 1 that the computed strain energy density follows the experimental data well.

### 4.1. An Amended Non-Gaussian Model for Stress-Softening and Residual Strain Effects

Encouraged by the accuracy of the predicted results obtained from the equivalent strain energy density given by Equation (16), we shall next take its derivative with respect to the amount of stretch [12] to obtain the corresponding material Cauchy stress-stretch virgin material constitutive equation of the form:

$$\text{T}=(1-f)ℵ\text{B}+\text{B}\frac{2f}{3}\left(A_{1}+\frac{2A_{2}}{3}(I_{1i}-3)\right){\text{a}}_{\text{i}}⊗{\text{a}}_{\text{i}}-p1$$ (17)

in which T is the Cauchy stress, B is the left Green–Cauchy deformation tensor, *p* is a hydrostatic pressure and ℵ denotes the material response function, given as [11]:

$$ℵ\equiv \frac{\mu }{3\lambda_{r}}\left[\beta +\frac{1}{N_{8}}\left(\frac{1}{\lambda_{r}}-\frac{1}{\beta \left(1-\lambda_{r}^{2}-\frac{2\lambda_{r}}{\beta }\right)}\right)\right]$$ (18)

To characterize the stress-softening effect, as well as residual strains, we use the material model introduced in [14,15] in which:

$$\begin{array}{cc} \tau_{k}=[(1-f)ℵ\lambda_{k}^{2}+\frac{2f}{3}\lambda_{k}^{2}\left(A_{1}+\frac{2A_{2}}{3}(I_{1i}-3)\right)x_{ki}^{2}-p+\frac{\mu \lambda_{k}}{2C}f_{k}(\lambda_{1},\lambda_{2},\lambda_{3})]e^{-b\sqrt{\left(M-m)(\frac{m}{M}\right)}}, & k=1,2,3(\text{no sum}) \end{array}$$ (19)

where:

$$f_{k}(\lambda_{1},\lambda_{2},\lambda_{3})=\frac{\partial \overset{3}{\underset{a=1}{\sum }}{\left(\lambda_{\text{max}a}^{n}-\lambda_{a}^{n}\right)}^{2}}{\partial \lambda_{k}}$$ (20)

Here, *C* is a positive material constant, *b* is a dimensionless material softening parameter, *n* is a fitting parameter that, in general, takes the value of one, λ*_a_* represents the principal stretches and *λ*_max_ *_a_, a* = 1, 2, 3 are the maximum values of the principal stretches at which unloading begins on the primary loading path.

Thus, the Cauchy stress-stretch equivalent material model components for the virgin material are obtained from Equation (17) as:

$$T_{k}=(1-f)ℵ\lambda_{k}^{2}+\frac{2f}{3}\lambda_{k}^{2}\left(A_{1}+\frac{2A_{2}}{3}(I_{1i}-3)\right)x_{ki}^{2}-p$$ (21)

Eliminating the pressure, *p*, from Equation (21) yields:

$$T_{j}-T_{k}=\left\{\left(1-f)ℵ+\frac{2f}{3}\left(A_{1}+\frac{2A_{2}}{3}(I_{1i}-3)\right)\{x_{ji}^{2}-x_{ki}^{2}\right\}\right\}(\lambda_{j}^{2}-\lambda_{k}^{2})$$ (22)

The corresponding constitutive equation for a non-monotonous stress-softened material model is provided by the following equation:

$$\tau_{j}-\tau_{k}=\left[\left\{\left(1-f)ℵ+\frac{2f}{3}(A_{1}+\frac{2A_{2}}{3}(I_{1i}-3))\{x_{ji}^{2}-x_{ki}^{2}\right\}\right\}(\lambda_{j}^{2}-\lambda_{k}^{2})+\frac{\mu }{2C}(\lambda_{j}f_{j}(\lambda_{1},\lambda_{2},\lambda_{3})-\lambda_{k}f_{k}(\lambda_{1},\lambda_{2},\lambda_{3}))\right]e^{-b\sqrt{\left(M-m)(\frac{m}{M}\right)}}$$ (23)

where *j* ≠ *k* = 1*,* 2*,* 3 (no sum). For simple extension homogeneous deformation, state *m* is given as:

$$m=\sqrt{\lambda^{4}+2\lambda^{-2}}$$ (24)

thus, the relative chain stretch, λ*_r_*, becomes:

$$\lambda_{r}=\sqrt{\frac{1}{3N}(\lambda^{2}+2\lambda^{-1})}$$ (25)

Before we assess the accuracy achieved by our proposed energy material model Equation (16), we shall next consider another material model that is based on the pseudo-elasticity theory.

### 4.2. Pseudo-Elastic Model for Stress-Softening and Permanent Set Effects

Here, we modify the pseudo-elastic material model proposed by Dorfmann and Ogden in [16] and consider energy expression Equation (16) to derive the corresponding unloading stress-stretch equations:

$$\tau_{j}-\tau_{k}=\left[\left\{\left(1-f)ℵ+\frac{2f}{3}\left(A_{1}+\frac{2A_{2}}{3}(I_{1i}-3)\right)\{x_{ji}^{2}-x_{ki}^{2}\right\}\right\}(\lambda_{j}^{2}-\lambda_{k}^{2})+(1-\eta_{2})(\nu_{1}\lambda_{j}^{2}-\nu_{2}\lambda_{k}^{2})\right]\eta_{1}$$ (26)

which describe Mullins and residual strain effects in which:

$$\eta_{1}=1-\frac{1}{r_{1}}\left(\text{tanh}\left[\frac{W_{\max}-W_{T}}{\mu m_{1}}\right]\right)$$ (27)

$$\eta_{2}=\frac{1}{r_{1}\text{tanh}[1]}\text{tanh}\left[{\left(\frac{W_{T}}{W_{\max}})\right)}^{\alpha }\right]$$ (28)

$$\nu_{1}=\nu_{2}\left(1-\frac{1}{r_{2}}\text{tanh}[10(\lambda_{\max}-1)]\right),$$ (29)

$$\alpha =\frac{1}{10}\left(3+\frac{8W_{\max}}{5\mu }\right)$$ (30)

wherein *W*_max_ = *W_T_* (λ_max_), *ν*_2_ = *γµ* and *m*_1_, *r*_1_, *r*_2_ and *γ* are material parameters. Of course, the uniaxial engineering stress-stretch relation, *σ*, for an incompressible material, as a function of the equivalent form of the Cauchy stress, is obtained from the following equation:

$$\sigma ={\text{TF}}^{-1}$$ (31)

We next use the aforementioned material models to predict uniaxial extension experimental data of human vaginal tissue, mice skin, two suture materials, tracheal and brain human tissue samples.

## 5. Models Comparison With Experimental Data

**Figure 2.**
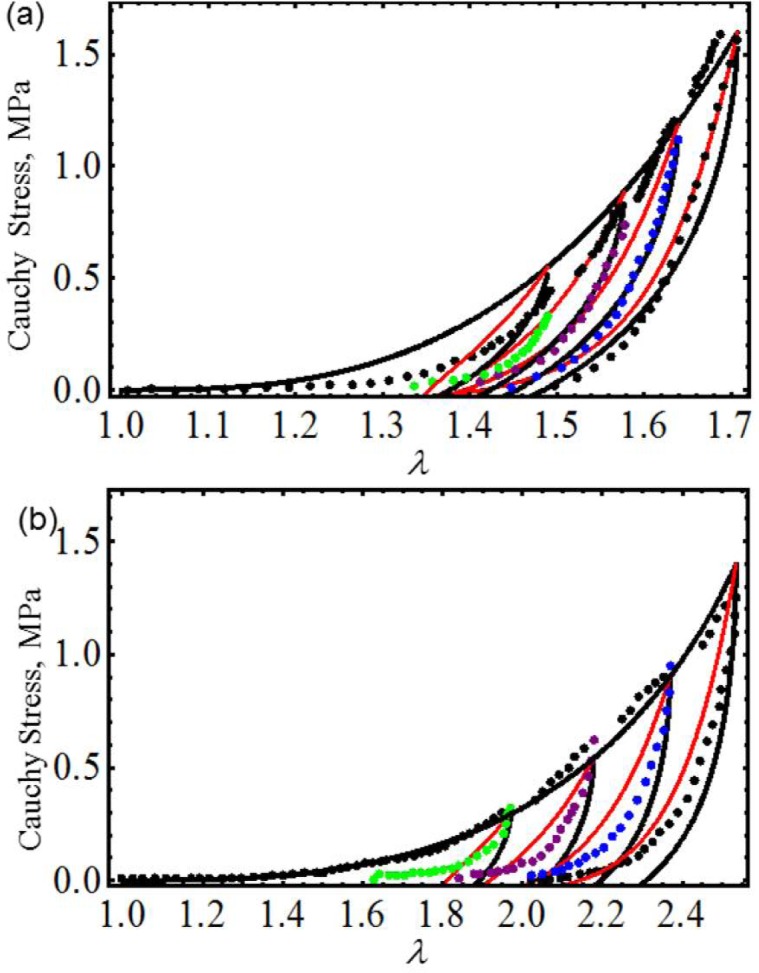
Cauchy stress-stretch data collected from human vaginal tissue compared with theoretical predictions. (**a**) Longitudinal samples of specimen I. (**b**) Transverse samples of specimen I. Here, the solid black lines describe the theoretical predictions obtained from our proposed model Equation (23), the red solid lines are predictions computed from pseudo-elastic material model Equation (26), while the color dots represent the experimental data adapted from [17].

We first start by considering uniaxial extension experimental data collected from samples of vaginal tissue subjected to loading and unloading cyclic tests along the longitudinal and transverse axes of the biological tissue samples [17]. Figure 2 shows the computed loading and unloading engineering stress curves in which stress-softening and permanent set effects are considered. Notice that, in both cases, our proposed equivalent material models not only predict the samples’ stiffness well at low stretch values, but also, they capture stress-softening and permanent set effects. In this case, the material constants used to best fit the experimental data by using the Dorfmann and Ogden material model have the value of *c* = −0.0284 J/m^3^, *m*_1_ = 1.8, *r*_1_ = 1.0001, *r*_2_ = 0.1 and *γ* = 0.3, for the longitudinal and transverse samples of specimen I. The material parameters, *μ*, *N*, *A*_1_, *A*_2_, *b*, *C* and *f*, of Equation (23) are listed in Table 1. Here, the solid black lines describe the theoretical predictions obtained from our proposed model Equation (23), the red solid lines are theoretical predictions computed from Dorfmann and Ogden pseudo-elastic material model Equation (26), while the color dots represent experimental data.

**Figure 3.**
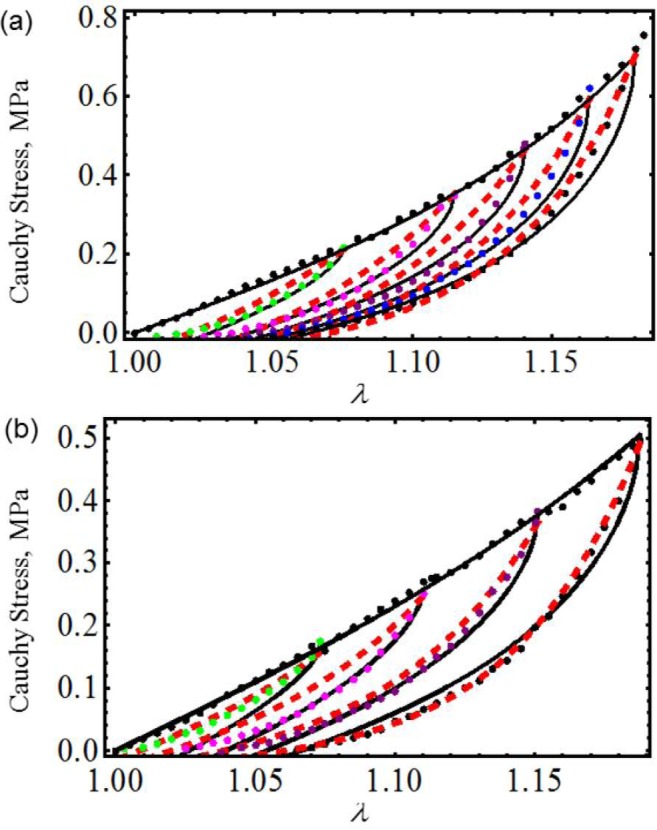
Cauchy stress-stretch data for mice skin compared with the theoretical predictions. The material parameter values used to obtain the theoretical predictions are summarized in Table 1. (**a**) Male skin; (**b**) female skin. The solid black lines represent the theoretical predictions obtained from Equation (23); the dashed red lines are predictions computed from Equation (26), while the color dots describe experimental data adapted from [18].

We next use experimental data collected from cyclic loading and unloading of 18-month male and female mice skin [18]. Figure 3 illustrates that theoretical predictions obtained from both material models closely follow experimental data. Here, *c* = 0.0994 J/m^3^, *m*_1_ = 0.0525, *r*_1_ = 1.00001, *r*_2_ = 0.55 and *γ* = 0.55 for male mouse and *c* = 0.7741 J/m^3^, *m*_1_ = 0.05, *r*_1_ = 1.001, *r*_2_ = 0.04 and *γ* = 0.55 for female mouse. The material constants of Equation (23) used to fit experimental data are summarized in Table 1. Once again, in Figure 3, the solid black and the red dashed lines represent theoretical predictions computed from Equations (23) and (26), respectively, while the color dots describe experimental data. Similar accuracy was achieved by Ehret and Itskov by using a thermodynamically consistent dissipative model to describe the softening phenomena in anisotropic materials [19]. However, their model requires the determination of nine material constants in each case, to capture softening and residual strain effects, while the material model described by Equation (23) only needs the determination of seven material parameters.

**Figure 4.**
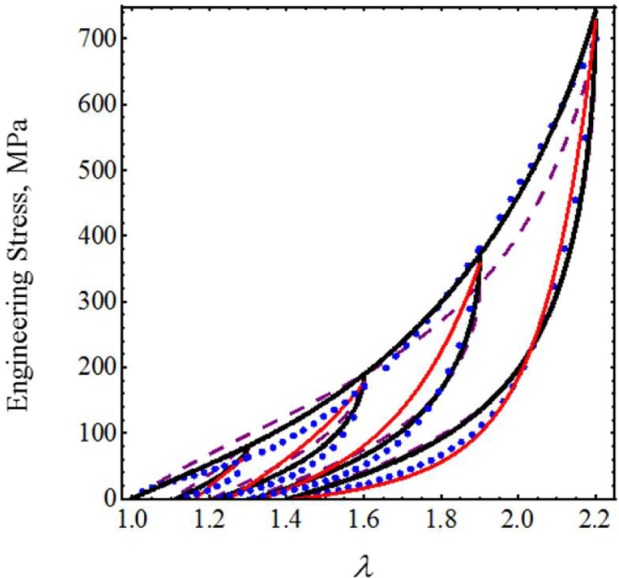
Engineering stress-stretch data for PGC25 3-0 sutures compared with theoretical predictions. The material parameter values used to obtain theoretical predictions from Equation (19) are summarized in Table 1, while the values of *c* = −27.5616 J/m^3^, *m*_1_ = 1.65, *r*_1_ = 1.1, *r*_2_ = 0.6 and *γ* = 1 were used in Equation (26).

**Figure 5.**
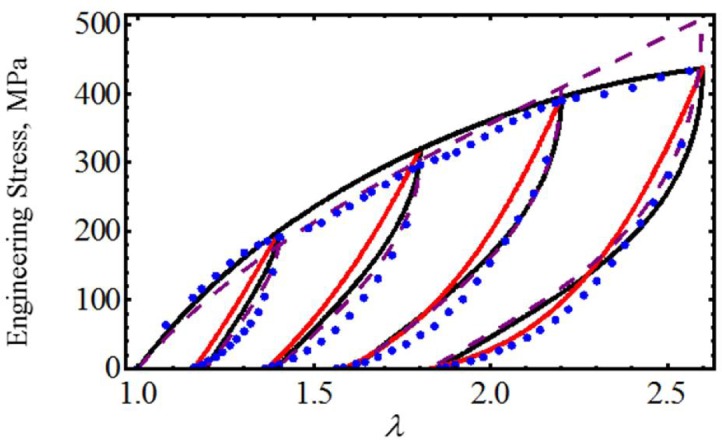
Engineering stress-stretch data for polypropylene sutures compared with theoretical predictions. The material parameter values used to obtain theoretical predictions from Equation (19) are summarized in Table 1, while the values of *c* = −78.5996 J/m^3^, *m*_1_ = 0.85, *r*_1_ = 1.0001, *r*_2_ = 0.35 and *γ* = 0.975 were used in Equation (26).

To further assess the accuracy of our proposed equivalent energy material model, we now use cyclic loading-unloading uniaxial stress-stretch data from poly(glycolide-co-caprolactone) (PGC25 3-0) and polypropylene suture material samples collected from an Instron tensile machine model 3365 with a maximum cell load capacity of 1.6 kN [20]. As we can see from Figures 4 and 5, the predicted stress-stretch curves computed from Equations (19) (solid black lines) and Equation (26) (red solid lines), to characterize the mechanical response of both suture material samples, describe the qualitative and quantitative behavior exhibited by the experimental data well (blue dots). In fact, our theoretical predictions are close to those reported in Figures 7 and 9 (dashed purple lines) of [20] in which an amended isotropic, hyperelastic non-Gaussian Arruda–Boyce material model was used. The material constants used to best fit the experimental data are listed in Table 1.

**Figure 6.**
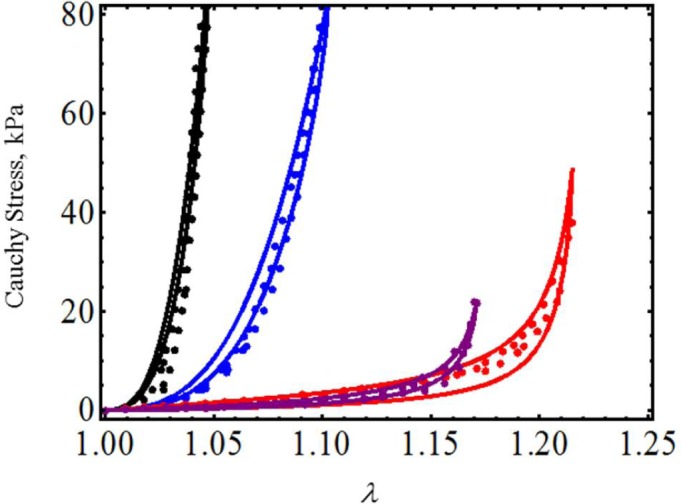
Cauchy stress-stretch data for specimens of the mucosa and submucosa human tracheal membrane. The material parameter values used to obtain the theoretical predictions are summarized in Table 2. Here, the solid black, blue, purple and red lines represent theoretical predictions, while the color dots describe the experimental data obtained from the CAM, CSM, ASM, and AAM mucosa and submucosa membranes. The experimental data were adapted from [21].

We next model the material behavior of human tracheal specimens by considering uniaxial test experimental data collected by Teng *et al*. in [21] in the circumferential and axial directions of the mucosa and submucosa membrane (CSM and ASM) and in the axial and circumferential directions of the adventitial membrane (AAM, CAM). We selected these experimental data in an attempt to characterize tracheal muscle and its surrounding connective tissues, which will help physicians to understand its process maturation and its related functional evolution [22]. Here, we only use Equations (17) and (19) to predict the experimental data, shown by the color dots in Figure 6, qualitatively and quantitatively. In Figure 6, the solid black, blue, purple and red lines represent, respectively, the theoretical predictions of the CAM, CSM, ASM and AAM mucosa and submucosa membrane material data. Table 2 exhibits the specimens’ predicted material shear modulus, as well as the material constants used to fit the experimental data. It is clear from Figure 6 that the experimental data and theoretical predictions agree well, even though the maximum amount of deformation stretch experienced by the specimens varies from 1.048 < λ_max_ < 1.215.

**Figure 7.**
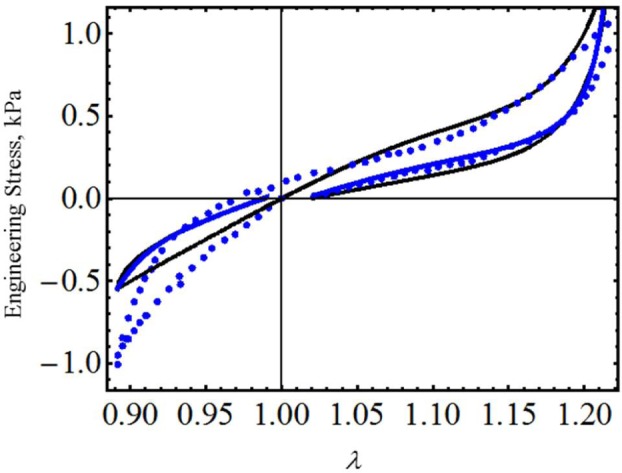
Engineering stress-stretch data for samples of brain tissue harvested from the frontal lobe in the sagittal direction compared with the theoretical predictions. The material parameter values used to obtain the theoretical predictions from Equation (19) are summarized in Table 3, while the values of *c* = −0.8263 J/m^3^, *m*_1_ = 0.095, *r*_1_ = 1.001, *r*_2_ = 0.8 and *γ* = 0.1 were used in Equation (26). The experimental data were adapted from [23].

**Figure 8.**
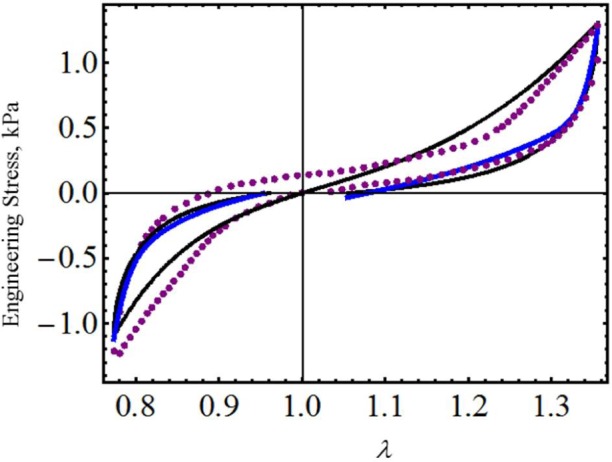
Engineering stress-stretch data for samples of brain tissue harvested from the occipital lobe in the frontal direction compared with the theoretical predictions. The material parameter values used to obtain the theoretical predictions from Equation (19) are summarized in Table 3, while the values of *c* = −0.0205 J/m^3^, *m*_1_ = 0.006, *r*_1_ = 2.5, *r*_2_ = 1.8 and *γ* = 0.1 were used in Equation (26). The experimental data were adapted from [23].

As a final example, let us consider the experimental data collected from human brain tissue that exhibits Mullins and residual strain effects, which are qualitatively similar to that observed in filled elastomers. Experimental data plotted in Figures 7 and 8 were obtained from two tests, one involving compression and subsequent tension (specimens of white matter harvested from the frontal lobe in the sagittal direction) and another involving tension and subsequent compression (specimens of white matter harvested from the occipital lobe in the frontal direction) [23]. We next use derived equivalent constitutive material model Equations (19) and (26) to predict both compression and tension tests. Theoretical simulations obtained from Equations (19) and (26) are illustrated in Figures 7 and 8 by the black and the blue solid lines, respectively. The corresponding material constants for both tests are summarized in Table 3. One must notice that both material models Equations (19) and (26) capture experimental stress-softening and permanent set effects well. In fact, the model described by Equations (17) and (19) closely follows the experimental data (black dots) collected from the occipital lobe in the frontal direction and shows some discrepancies with respect to data collected from the frontal lobe in its frontal direction. These computed predictions have better agreement with experimental data than those obtained from the material model developed in [23] in spite of having neglected the material viscoelastic effects.

## 6. Conclusions

In this paper, we have used the rule of mixtures to develop material models that are based on the equivalent representation form of the strain energy density of hyperelastic materials. This equivalent representation form of the strain energy density follows the idea of finding the isotropized energy form of polymeric materials reinforced with carbon nanotubes. Here, we adopted that isotropized energy form and used the non-Gaussian amended strain energy density form Equation (8) in combination with the rule of mixtures to develop an equivalent strain energy density expression that captures the anisotropic energy contribution during the loading and unloading of biocompatible materials subjected to uniaxial stresses. Furthermore, we have identified *f* as the equivalent anisotropic volumetric fraction contribution. We have used experimental data collected from samples of vulcanized natural rubber in which the percentage of the isotropic and anisotropic strain energy density contributions were known, and then, we have applied our derived equivalent strain energy density equation to verify the accuracy of our model by plotting the strain energy density *versus* the amount of stretching. We have found that our model describes the qualitative and quantitative experimental data well. In fact, we have observed that when the value of *f* = 25%, the theoretical predictions obtained from our equivalent strain energy density match nicely with the data exhibited in Figure 1. We recall that this percentage value of *f* represents the percentage of the anisotropic contribution of the rubber material samples when subjected to uniaxial loads [9]. Based on these results, we next developed the corresponding stress-stretch relations that take into account the stress-softening and residual strain effects that are commonly exhibited by biological materials. Then, we have used these to predict the experimental data collected from human vaginal tissues, mice skin, poly(glycolide-co-caprolactone) (PGC25 3-0) and polypropylene material samples and data collected from human tracheal and brain tissues. In most of the experimental data considered here, we have found that theoretical predictions obtained from Equations (19) and (26) describe the stress-softening and residual strain effects of material samples subjected to uniaxial stresses well. Based on these results, we believe that the accuracy achieved by these material models could be related to the inclusion of the amended term in the non-Gaussian strain energy density equation, since, in accordance, with the values of the chain number of links, *N*, listed in Tables 1 and 3, the amended term of the strain energy density, given by expression Equation (8), influences the qualitative and quantitative behavior of the computed stress-stretch theoretical predictions.

## Figures and Tables

**Table 1.** Material constants used to fit experimental data.

Material samples	*μ* (MPa)	*N*	*A*_1_ (MPa)	*A*_2_ (MPa)	*b*	*C* (MPa)	*f* (%)
Vaginal tissue (longitudinal axis)	0.085	3.25	−6.5	70	1.3	0.7	0.2
Vaginal tissue (transverse axis)	0.085	3.25	−6.5	3.93	1.3	0.7	0.2
Male mouse skin	0.95	1.082	0	30	2.8	0.98	9.0
Female mouse skin	0.77	1.18	0	20	2.55	1.2	9.0
PGC25 suture material	100	2.35	0	1300	0.95	0.008	10
Polypropylene suture material	300	50.5	−7500	−2100	0.6	0.0024	1.35

**Table 2.** Material constants used to fit the human tracheal experimental data.

Material samples	*μ* (kPa)	*N*	*A*_1_ (MPa)	*A*_2_ (MPa)	*b*	*C* (kPa)	*f* (%)
Circumferential mucosa and submucosa membrane (CSM)	5	1.029	0	2, 500	1.3	2.1	0.8
Axial mucosa and submucosa membrane (ASM)	5	1.029	0	15	1.3	2.1	0.8
Circumferential adventitial membrane (CAM)	10	1.045	0	35, 000	1	3.4	0.6
Axial adventitial membrane (AAM)	10	1.045	0	1.5	1	3.4	0.6

**Table 3.** Material constants used to fit the brain tissue experimental data.

Material samples	*μ* (kPa)	*N*	*A*_1_ (kPa)	*A*_2_ (kPa)	*b*	*C* (kPa)	*f* (%)
Frontal lobe (sagittal direction: tension)	2.6	1.065	−150	−3000	2.7	4.6	1
Frontal lobe (sagittal direction: compression)	2.6	1.065	−150	−3000	2.5	0.6	1
Occipital lobe (frontal direction: tension)	2.65	2.5	−350	650	2.7	3.8	0.93
Occipital lobe (frontal direction: compression)	2.65	2.5	−350	650	2.7	3.8	0.93
